# ICTV Virus Taxonomy Profile: *Filoviridae*

**DOI:** 10.1099/jgv.0.001252

**Published:** 2019-04-25

**Authors:** Jens H. Kuhn, Gaya K. Amarasinghe, Christopher F. Basler, Sina Bavari, Alexander Bukreyev, Kartik Chandran, Ian Crozier, Olga Dolnik, John M. Dye, Pierre B. H. Formenty, Anthony Griffiths, Roger Hewson, Gary P. Kobinger, Eric M. Leroy, Elke Mühlberger, Sergey V. Netesov (Нетёсов Сергей Викторович), Gustavo Palacios, Bernadett Pályi, Janusz T. Pawęska, Sophie J. Smither, Ayato Takada (高田礼人), Jonathan S. Towner, Victoria Wahl, ICTV Report Consortium

**Affiliations:** ^1^​ NIH/NIAID Integrated Research Facility at Fort Detrick, (IRF-Frederick), Frederick, MD 21702, USA; ^2^​ Washington University School of Medicine, St. Louis, MO 63110, USA; ^3^​ Center for Microbial Pathogenesis, Georgia State University, Atlanta GA 30303, USA; ^4^​ United States Army Medical Research Institute of Infectious Diseases, Frederick, MD 21702, USA; ^5^​ The University of Texas Medical Branch, Galveston, TX 77555, USA; ^6^​ Albert Einstein College of Medicine, Bronx, NY 10461, USA; ^7^​ Integrated Research Facility at Fort Detrick, Clinical Monitoring Research Program Directorate, Frederick National Laboratory for Cancer Research supported by the National Cancer Institute, Frederick, MD 21702, USA; ^8^​ Institute of Virology, Philipps University Marburg, 35043 Marburg, Germany; ^9^​ World Health Organization, CH-1211 Geneva, Switzerland; ^10^​ Department of Microbiology and National Emerging Infectious Diseases Laboratories, Boston University School of Medicine, Boston, MA 02118, USA; ^11^​ Public Health England, Porton Down, Wiltshire, SP4 0JG Salisbury, UK; ^12^​ Université Laval, Québec City, QC G1V 0A6, Canada; ^13^​ Infectious Diseases and Vectors Unit, Institut de Recherche pour le Développement, 911 Av Agropolis, 34394 Montpellier, France; ^14^​ Novosibirsk State University, Novosibirsk, Novosibirsk Oblast, 630090, Russia; ^15^​ National Biosafety Laboratory, National Public Health Center, Budapest, Hungary; ^16^​ National Institute for Communicable Diseases of the National Health Laboratory Service, 2131 Sandringham-Johannesburg, Gauteng, South Africa; ^17^​ Defence Science and Technology Laboratory, Porton Down, Salisbury, Wiltshire SP4 0JQ, UK; ^18^​ Division of Global Epidemiology, Hokkaido University Research Center for Zoonosis Control, 001-0020 Sapporo, Japan; ^19^​ Viral Special Pathogens Branch, Centers for Disease Control and Prevention, Atlanta, GA 30333, USA; ^20^​ National Biodefense Analysis and Countermeasures Center, Frederick, MD 21702, USA

**Keywords:** *Filoviridae*, filovirus, ICTV Report, ebolavirus, marburgvirus, taxonomy

## Abstract

Members of the family *Filoviridae* produce variously shaped, often filamentous, enveloped virions containing linear non-segmented, negative-sense RNA genomes of 15–19 kb. Several filoviruses (e.g., Ebola virus) are pathogenic for humans and are highly virulent. Several filoviruses infect bats (e.g., Marburg virus), whereas the hosts of most other filoviruses are unknown. This is a summary of the International Committee on Taxonomy of Viruses (ICTV) Report on *Filoviridae*, which is available at www.ictv.global/report/filoviridae.

## Virion

**Fig. 1.**
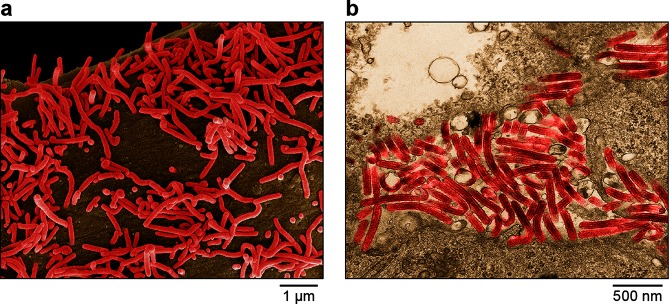
Electron microscopic images of Marburg virus particles budding from infected Vero E6 cells, (a) scanning EM, (b) transmission EM. Images are colourized for clarity. Courtesy of John G. Bernbaum and Jiro Wada, IRF-Frederick.

Virions are enveloped and diverse in shape and can appear as branched, toroid, U- or 6-shaped, and long filamentous forms (Table 1, Fig. 1). Virions contain ribonucleoprotein (RNP) complexes composed of genomic RNA and, typically, the structural proteins nucleoprotein (NP), polymerase co-factor (VP35), transcriptional activator (VP30), RNP-associated protein (VP24) and RNA-dependent RNA polymerase (L). The matrix protein (VP40) forms a regular layer beneath the viral envelope. Surface spikes formed by glycoproteins (GP_1,2_) are approximately 7 nm in diameter and cover the virion surface at approximately 10-nm intervals [1–3]. Some filoviruses do not have discernable glycoproteins and may have different RNP complexes [4].

**Table 1.** Characteristics of members of the family *Filoviridae*

**Typical member:**	**Marburg virus (DQ217792), species *Marburg marburgvirus*, genus *Marburgvirus***
Virion	Enveloped, variously shaped but predominantly filamentous, typically with a single nucleocapsid
Genome	Approximately 15–19 kb of linear, negative-sense, non-segmented RNA
Replication	Antigenomic RNA is a replication intermediate. The genome and antigenome form ribonucleoprotein complexes, which serve as templates
Translation	From multiple 5′-capped and 3′-polyadenylated mRNAs
Host range	Primates (e.g., ebolaviruses, marburgviruses), bats (e.g., marburgviruses), domestic pigs (e.g., Reston virus) and probably fish (e.g., striaviruses, thamnoviruses) become naturally infected
Taxonomy	Realm *Riboviria*, phylum *Negarnaviricota*, subphylum *Haploviricotina*, class *Monjiviricetes*, order *Mononegavirales*; family includes multiple genera

## Genome

**Fig. 2.**
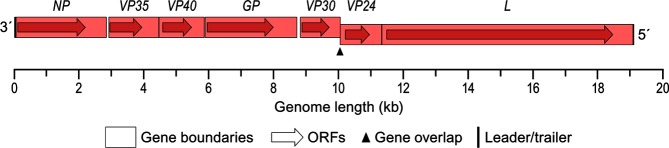
Schematic representation of the genome organization of Marburg virus. Courtesy of Jiro Wada, IRF-Frederick.

Filovirus genomes are approximately 15–19 kb (Fig. 2) without a 5′-cap or 3′-poly(A). Terminal leader and trailer sequences contain the replication and transcription promoters. Marburgvirus genomes contain seven separate, continuous open reading frames (ORFs) flanked by 3′- and 5′-terminal non-coding regions that contain transcription initiation and termination sites. These ORFs encode the virion structural proteins. Cuevavirus and ebolavirus genomes encode homologues of the marburgvirus structural proteins. However, the marburgvirus *GP* gene encodes only GP_1,2_, whereas the primary expression product of cuevavirus and ebolavirus *GP* gene transcription is a soluble glycoprotein. Co-transcriptional editing is used to express GP_1,2_ and additional non-structural proteins. Striaviruses and thamnoviruses encode some, but not all, marburgvirus protein homologues and several proteins of unknown function [4, 5].

## Replication

Virus proteins are translated from mRNAs that are synthesized by successive, polar transcription from RNP complexes containing genomic RNA. Replication occurs in the cytoplasm through the synthesis of RNP complexes containing antigenomes that are templates for genomic RNA production. Replication and transcription enzymes include L and VP35. VP30 serves as a transcription enhancer for ebolaviruses and probably cuevaviruses, but its function in marburgvirus infection is less defined. Virion assembly, including acquisition of the GP_1,2_-containing lipid envelope, occurs by VP40-mediated budding at the plasma membrane [4, 5].

## Taxonomy

Filoviruses form a family in the haploviricotine order *Mononegavirales*. Within this order, filoviruses are most closely related to members of the families *Paramyxoviridae*, *Pneumoviridae* and *Sunviridae*. The family *Filoviridae* includes multiple genera for viruses that differ in geographic and host range and genomic organization.

## Resources

Full ICTV Report on the family *Filoviridae*: www.ictv.global/report/filoviridae.
